# Supplementary material for: Extraction and characterization of collagen and gelatin from body wall of sea cucumbers Stichopus horrens and Holothuria arenicola
Source: PeerJ. 2024 Oct 10;12:e18149. doi: 10.7717/peerj.18149 (PMC11471148; doi:10.7717/peerj.18149)
Supplement: Supplemental Information 2 [file peerj-12-18149-s002.pdf]

Uncropped Gels/Blots Required

Submission reporting Western blot experiments must be accompanied by photos of the full-length blots, bearing appropriate molecular weight ladders.

Each lane must be appropriately labeled, and the molecular weights of all of the ladder components must be included.

For each Western blot experiment, the same blot must be used for the protein(s) of interest and the loading controls.

As per PeerJ policy, provide your full-length uncropped gels/blots (Figure 4).

- Upload the images (preferably as a single compressed zip file) to the Supplemental Files.
- Molecular weight ladders must be visible.
- Please ensure you include all replicates.

We can only provide images of the cropped gel that has been prepared, as a complete image of the entire gel is not available. Due to the utilization of a single SDS-PAGE gel for eight protein samples, each loaded individually by different researchers, it was not feasible for me to retain data from other contributors. Therefore, each researcher mounted their respective sample onto the gel."

Reply:

Dear Editor,

Thank you for your message regarding the requirements for Western blot experiments.

Regarding your request for uncropped gels/blots, we regret to inform you that we are unable to provide full-length images of the gel. Our methodology involved the utilization of a single SDS-PAGE gel for eight protein samples, each loaded individually by different researchers. As a result, we only possess images of the cropped gel, and a complete image of the entire gel is not available.

We understand the importance of reproducibility in scientific research, and we apologize for any inconvenience caused by our inability to provide uncropped gel/blot images.

We will ensure that all relevant details are included in the submitted materials. If you need further clarification or assistance, please let us know.

Thank you for your understanding and cooperation.

Best regards,

Noora Barzkar

Borneo Marine Research Institute, Higher Institute Centers of Excellence, Universiti Malaysia Sabah, Kota Kinabalu 88450, Sabah, Malaysia

Email address: noora.barzkar@ums.edu.my or noora.barzkar@gmail.com
